# A Review on Various Uses of N-Acetyl Cysteine 

**DOI:** 10.22074/cellj.2016.4872

**Published:** 2016-12-21

**Authors:** Vida Mokhtari, Parvaneh Afsharian, Maryam Shahhoseini, Seyed Mehdi Kalantar, Ashraf Moini

**Affiliations:** 1 Department of Molecular Cytogenetics, Research and Clinical Center for Infertility, University of Medical Sciences, Yazd, Iran; 2 Department of Genetics, Reproductive Biomedicine Research Center, Royan Institute for Reproductive Biomedicine, ACECR, Tehran, Iran; 3 Department of Endocrinology and Female Infertility, Reproductive Biomedicine Research Center, Royan Institute for Reproductive Biomedicine, ACECR, Tehran, Iran; 4 Department of Obstetrics and Gynecology, Roointan-Arash Hospital, Tehran, Iran

**Keywords:** N-Acetyl Cysteine, Antioxidant, Oxidative Stress

## Abstract

N-acetyl cysteine (NAC), as a nutritional supplement, is a greatly applied antioxidant *in vivo* and *in vitro*. NAC is a precursor of L-cysteine that results in glutathione elevation biosynthesis. It acts directly as a scavenger of free radicals, especially oxygen radicals. NAC is a powerful antioxidant. It is also recommended as a potential treatment option for different disorders resulted from generation of free oxygen radicals. Additionally, it is a protected and endured mucolytic drug that mellows tenacious mucous discharges. It has been used for treatment of various diseases in a direct action or in a combination with some other medications. This paper presents a review on various applications of NAC in treatment of several diseases.

## Introduction

N-acetyl cysteine (NAC), as a safe and inexpensive medication, is commercially accessible since long-time ago (1). This drug is not found in natural sources, although cysteine is present in some meals like chicken and turkey meats, garlic, yogurt, and eggs (2). NAC is a well-tolerated mucolytic drug that moderates clinging mucous secretions and enhances glutathione S-transferase activity. During oral administration, deacetylation reaction of NAC happens while passing along the small intestine as well as liver, thus its bioavailability is decreased to 4-10%. NAC stimulates glutathione biosynthesis, promotes detoxification, and acts directly as a scavenger of free radicals. It is a powerful antioxidant and a potential treatment option for diseases characterized by the generation of free oxygen radicals (3). Studies have shown no maternal or fetal harmful effects of NAC treatment. This nutritional supplement is an excellent source of sulphydryl groups. NAC prevents apoptosis and oxygen related genotoxicity in endothelial cells by increasing intracellular levels of glutathione and decreasing mitochondrial membrane depolarization (4). The critical antioxidant power of NAC is due to its role as a precursor of glutathione, which is one of the most important naturally occurring antioxidants (5). NAC combination with vitamin E, or vitamins A+E, as well as essential fatty acids considerably reduce reactive oxygen species (ROS), leading to pregnancy rate improvement (6). Studies have indicated that preserving impact of NAC against the toxicity of chemicals is due to its dual role as a nucleophile and as a -SH donor (7). In this study by reviewing literatures, various applications of NAC in treatment of some diseases are highlighted. 

### Polycystic ovary syndrome

Polycystic ovary syndrome (PCOS) is one of the most common endocrine glands-related diseases affecting 5-10% of reproductive-age women (8). This syndrome is considered as the most common cause of anovulatory infertility. PCOS is also associated with pregnancy complications such as recurrent pregnancy loss (RPL). Different studies report the prevalence of PCOS in women with a history of RPL in a wide range of 10-82%. Findings show that 70.7% of PCOS women with a previous RPL had thrombophilic disorders. In addition, prevalence of protein C deficiency is significantly higher in PCOS patients compared to the non-PCOS women (9). 

The results of a study showed that women with PCOS have a high prevalence of metabolic syndrome and its individual components (obesity, hypertension, glucose intolerance and triglycerides), particularly decreased high density lipoprotein cholesterol (10). In another study, women and their relatives with PCOS had an increased prevalence of diabetes commonly in mother’s side of the family (11). 

As the first medication option, clomiphene citrate (CC) is applied for the induction of ovulation in PCOS women. A collection of published results for treatment with CC showed a pregnancy rate and a miscarriage rate of 36 and 20.4%, respectively. One of the frequently determined problems of this treatment is resistance to CC in up to 40% of PCOS patients. NAC is a mucolytic drug with insulin-sensitizing properties that has been used successfully as a supporting therapy in subjects with CC-resistant PCOS (8). Recent studies have shown that a combination of CC and NAC considerably increased both ovulation and pregnancy rates in women with CC-resistant PCOS. NAC has multiple biological effects, two of which are potentially and directly related to pregnancy rate improvement. NAC has mucolytic action, thus it can revoke the negative effect of CC on cervical mucus. Simultaneously, it has insulin sensitizing effect that could assist in issues related to PCOS. The negative influence of CC on cervical mucus can create a "hostile" environment for conception (1). 

Researchers evaluated the effect of NAC, known to resupply stores of the antioxidant glutathione, on insulin secretion and peripheral insulin resistance in subjects in association with PCOS. Moreover, treatment of hyperinsulinemic patients by NAC was found to tailor control parameters of glucose in them and consequently, their insulin levels and peripheral insulin sensitivity were reduced and increased, respectively. Therefore, the antioxidant effects of NAC may act as a therapeutic approach to improve the level of circulating insulin as well as insulin sensitivity in PCOS patients with hyperinsulinemia (12). 

### Premature birth and recurrent pregnancy loss

Premature birth is the most common reason of perinatal mortality and long-term unhealthiness in low-income countries (13). Inflammation, fetal infection, and previous preterm delivery are significant risk factors for preterm birth and neonatal brain injury (14). Rising infection with bacterial vaginosis during pregnancy is related to a risk factor for preterm delivery and low birth weight. Antimicrobial medical care is, although, not adequate for the prevention of preterm birth and the inflammatory as well as anti-inflammatory responses could make problem complex (3). NAC by having an anti-inflammatory outcome can affect human term and preterm labors. NAC restrains the inflammatory response with no respect whether infection is started before or after treatment initiation with the drug. Shahin et al. (3) concluded that in women with previous preterm birth and bacterial vaginosis, 0.6 g of NAC daily can be taken orally along with progesterone after week 16 of pregnancy to protect against preterm birth recurrence and improve neonatal outcome. 

RPL is defined as the occurrence of three or more consecutive pregnancy losses in the first or early second trimester of pregnancy (less than 20 weeks of gestation). It is one of the most common clinical problems in reproduction that a certain cause can be found in only 50% of cases (15). Many etiologies have been suggested for RPL (16). For example, molecular genetic background for RPL is being increasingly understood, and some polymorphisms associated with RPL have been reported. According to the research directed up to now, more than 40 gene products distinctively have been distinguished to be expressed in women with RPL compared to healthy women. These genes may have regulatory roles in establishing or maintaining normal pregnancy. In this manner, any nucleotide modifications in targeted genes may result in distinct expression and activity endangering general well-being during pregnancy. A recent study showed a relationship between c.179A>C mutation in the Bax promoter and RPL and also, two polymorphisms, namely c.90G>C and c.95G>A in exon 1, found among patients that can be considered as genetic factors making people susceptible to miscarriages (17). Findings of the other recent study revealed a new genetic relationship between occurrences of RPL and SULF1 gene mutation. SULFs are a protein family of arylendosulfatase. They act as post synthetic editors that can selectively release 6-O-sulfate groups from heparin sulfates, consequently changing the sulfation patterns of proteoglycans and binding site of many growth factors. With such unique regulatory activity, SULFs have an important role in many biological processes, such as angiogenesis, cell signaling and embryogenesis. In this gene family, SULF1 is expressed in the large number of embryonic and adult tissues, while it has an important role in viability and embryonic development (18). In a study conducted by some researchers, high rate of mutations in D-loop of mtDNA was observed in maternal blood, a fact that may have a direct or indirect role in inducing RPL. This outcome can be utilized as part of the RPL evaluation, planning conceivable medications for enhancing the results of assisted reproduction (19). Some evidence has shown that oxidative stress might be a contributory factor in RPL (16). One phenomenon that is known as a common patho-physiological pathway for various etiologies of RPL, can be placental oxidative stress. Amin concluded that NAC is a well-tolerated drug that could potentially be an effective treatment in patients with unexplained RPL. Administration of a combination of NAC and folic acid, in comparison with folic acid alone, is resulted in prolongation of a living pregnancy up to 20 weeks. In addition, combination of NAC and folic acid were also associated with a significant increase in the take-home baby rate, as compared to folic acid alone (4). 

### Acetaminophen toxicity

Acetaminophen administration is reported as the most common drug overdose in pregnancy. Acetaminophen readily crosses the placenta and in toxic doses can result in fetal hepatic necrosis, premature birth, spontaneous abortion, and fetal death (2). NAC is an amino acid that contains thiol group. It has been used for the treatment of acetaminophen toxicity (20). N-Acetyl-pbenzoquinonimine is a potent oxidative metabolite of acetaminophen, resulting in hepatotoxicity, if it is not reduced by glutathione. NAC is thought to affect through multiple mechanisms including replenishing glutathione, reducing N-acetyl-pbenzoquinonimine directly, and performing nonspecific hepatoprotective actions related to its antioxidant properties. This compound is used to treat acetaminophen poisoning throughout pregnancy (3). It is universally effective to prevent hepatotoxicity, if it is administrated within 10 hours of acetaminophen overdose (21). 

In addition, acetaminophen toxicity is a common cause of drug-induced hepatotoxicity in children and adults. NAC has been used for several decades and it has been proven as a counterpoison choice in treating acetaminophen-induced hepatotoxicity. There is considerable clinical evidence to support the fact that oral and intravenous NAC are equally effective in the prevention of hepatotoxicity. An important factor in evaluating the effectiveness of NAC is the timing of therapy commencement in relation to the administration. Patients that ingest a severe overdose and have NAC therapy started within 8 hours get well and have less than a 10% rate of occurrence of hepatotoxicity. They generally do not develop liver failure or death. Those patients who have chronically taken immoderate doses of acetaminophen over many hours and/or have NAC therapy started more than 8 hours after an acute overdose are at a risk of hepatotoxicity with approximately 8-50% incidence (22). 

Several recent studies have investigated the antioxidant properties of NAC in feto-placental metabolism (23,24). NAC appears to attenuate placental production of inflammatory cytokine interleukin 6 involved in placenta infection and inflammation in the pregnant rat model. A murine model demonstrated benefit of NAC in a complicated pregnancy, manipulated by infection and free radical production, suggesting the ability of NAC to restore maternal and fetal oxidative balance to reduce preterm birth caused by different factors such as acetaminophen toxicity (21). 

*In vitro*studies have shown that oxidative stress might serve as a signal to initiate and propagate the inflammatory process, resulting in apoptosis of placental tissues (6). Bloosesky et al. (25) showed that preventative effect of NAC could reduce fetal inflammatory cytokines in response to maternal lipopolysaccharide. Their results suggested that prophylactic NAC administered in pregnancies was associated with a risk of maternal infection/inflammation, likely protecting fetus from adverse inflammatory sequelae. 

### Administration of N-acetyl cysteine for infertile patients undergoing assisted reproductive

techniques Elgindy et al. (5) found that 1200 mg NAC (daily) supplementation, started with administration of human menopausal gonadotrophin till the day of human chorionic gonadotrophin, did not significantly increase pregnancy rate in intracytoplasmic sperm injection (ICSI) cycles using the long agonist protocol. NAC treatment was associated with insignificant decrease in granulosa cell apoptosis, as well as insignificant increase in fertilization rate and grade-one embryo formation. Larger-scale studies, possibly with higher doses and/or longer duration of NAC administration, should be performed to identify any significant effects. In addition, Cheraghi et al. (26) conducted a study to determine the effect of co-administration of NAC and metformin on ovulation induction in PCOS patients with ICSI cycle. They detected that co-administration of these two components has no benefit for ovulation induction in PCOS patients with ICSI cycle. In another study, Rizk et al. (27) investigated the effect of NAC on performance of CC in ovulation induction. They concluded that the combination of NAC with CC is an effective way for ovulation induction in young women undergoing ICSI cycles. 

### Chronic bronchitis

Chronic bronchitis is defined as the presence of chronic productive cough for more than three months in each of two sequential years. Therefore, an important goal in the treatment of chronic bronchitis is to decrease the frequency and duration of intensification, and to decrease symptoms in patients with aggravations. In some European countries, mucolytic drugs, particularly NAC may be used as an anti-inflammatory drug as well as an antioxidant (28,29). In these countries, it is believed that NAC can decrease the frequency of aggravations and improve symptoms in patients with chronic bronchitis. Recently, a comprehensive review in literature survey has concluded in the field of the effectiveness of any oral mucolytic drugs that a decline of aggravations, days of disability and days of antibiotic treatment was averagely determined in patients with chronic clogging pulmonary disease (30). 

### Ulcerative colitis

Ulcerative colitis is a chronic inflammatory disorder which multiple casual factors can affect it. Human colitis has many similar characteristics to acetic acid (AA)-induced colitis, as a reproducible and simple model. Studies have indicated that some signaling pathways contributing in cell apoptosis and growth, angiogenesis, redoxregulated gene expression, and inflammatory response can be affected by NAC (6,31,33). Therefore, NAC may not only protect against the direct detrimental impacts of oxidants, but also advantageously modify inflammatory events in colitis (34). The beneficial influences of NAC were related to the changes including: i. Softened colonic injury, ii. Decreased oxidative stress, iii. Lowered cell apoptosis, iv. Increased recovery of the injured colon, and v. Increased formation of the tight junction (6). 

### Liver cancer

Liver cancer is one of the most common lifethreatening malignancies, all over the world and up to now, there is no effective drug for the treatment of liver tumors (35). Although, interferon (IFN) is the most applied medication in chronic hepatitis and hepatocarcinoma, due to its immune response activation property and also regulation of differentiation and cell growth (36). NAC, as an enhancer of glutathione biosynthesis (37), is one of the frequently used antioxidant drugs for treatment of liver disorders (38,39). Cell culture and animal models have shown that NAC can preserve normal cells against toxicity of radiotherapy and chemotherapy, but not cancerous cells (37). Administration of NAC may play a role in treatment of some forms of cancer, while induced damages in DNA can be completely blocked by NAC (38). 

### Muscle performance

Investigations showed no effect of NAC on nonfatigued muscle, although after three minutes of repetitive contractions, it caused a considerably enhanced force output, up to approximately 15%. This means that NAC can improve muscle performance. This result is originated from the fact that oxidative stress plays a causal role in the fatigue process, since NAC is a scavenger of free radicals causing oxidative stress. It has been wellreported that infusion of NAC can be effective on enhancing the overall redox status *in vivo*. It has also been shown that NAC infusion could minimize the muscle fatigue (40). 

### Hemodialysis

Homocysteine (Hcy) is a sulphur-containing amino acid that is produced in body, by the metabolism of the methionine amino acid (41). Hcy level in patients with hemodialysis is associated with kidney-related disorders. However, in the treated hemodialysis patients, some studies have shown that NAC administration could affect plasma Hcy levels. There are some reports indicating that NAC, with an antioxidant property, has declined plasma Hcy level in the end stage renal disease (ESRD) patients undergoing hemodialysis. Although, lower dosage of NAC (for example, 600 mg/day for a period of one month) could not help to decrease Hcy plasma levels in these patients (42). 

### Asthma

Asthma is a chronic disorder associated with inflammation and immune cell infiltration of airways (43). Airway hyper-responsiveness (AHR) can be originated from consistent presence of inflammatory mediators and immune cells in airways. AHR is clinically determined with breathlessness, coughing and wheezing symptoms (44). Studies showed the preventive effect of NAC antioxidant on the AHR and steroid resistant accumulation of inflammatory cells in the airways of the animal model with acute exacerbation of asthma (45,47). 

### Alzheimer

Alzheimer disease (AD) is known as a multifactorial disease with many abnormalities in physiological, biochemical, and neurochemical point of view. Aging is the major risk factor for AD that coexists with other causes of cognitive decline, particularly vascular dementia (48). Some factors, such as mitochondrial dysfunction, abnormal protein aggregation, metal accumulation, inflammation and excitotoxicity play important roles in AD pathology. Although the relationship between these factors and development of AD is multidirectional, oxidative damage is considered as a common thread linking some of these factors (49). Results of different studies showed that lipoic acid (LA) and NAC decreased levels of oxidative and apoptotic markers via protection of mitochondrial function (50,53). Combination of both LA and NAC maximizes this protective effect suggesting that this may prevent mitochondrial decay associated with aging and age-related disorders such as AD. Antioxidant therapies based on LA and NAC seem promising since they can act on mitochondria, one key source of oxidative stress in aging and neurodegeneration (50). 

### Parkinson

Parkinson disease (PD) is a very prevalent neurodegenerative disorder caused by unknown deterioration of cells which generate dopamine in the pars compacta, a part of the substantial nigra located in the midbrain (54). In terms of pathogenesis, PD appears to be multi-factorial disorder, including environmental factors, acting on genetically vulnerable individuals when they are older (55,56). A wide range of both genetic and environmental factors have been proposed as contributing to the initiation and progression of PD, but aging is the single most important risk factor for this disorder and undoubtedly interferes in PD progression through its accumulative oxidative damage, decrease in antioxidant ability and impairment of mitochondrial bio-energetic capacity in the brain (57,58). Taking into account that most of PD patients experience accumulative oxidative damage, some clinical studies have demonstrated the controversial effect of some antioxidant administrations -such as NACon treatment of PD (59,61). Some improvements have been reported for systemic administration of NAC in animal models, such as: i. Enhancement of brain level of glutathione, ii. Reduction of oxidative damage-markers, iii. Enhancement of brain synaptic and non-synaptic brain mitochondrial complex I activities, and iv. Protection against dopamine-induced cell death (59). 

## Conclusion

A review on NAC literature shows that this agent is a safe and well-tolerated supplementary drug without any considerable side effects. It 16 is as an antioxidant with a free radical scavenger property, as important characteristic of this medical supplement. It has been used as a beneficial drug treatment for some disorders such as poly cystic ovary syndrome patients with CC resistance, preterm birth, acetaminophen toxicity, RPL, chronic bronchitis, ulcerative colitis, liver cancer, muscle performance, hemodialysis, asthma, Alzheimer and Parkinson. Although in some cases, such as improving pregnancy rate in ICSI cycles, NAC action is still unclear and further investigations are necessary. 
